# Commercially Available Natural Benzyl Esters and Their Synthetic Analogs Exhibit Different Toxicities against Insect Pests

**DOI:** 10.1038/s41598-018-26242-6

**Published:** 2018-05-21

**Authors:** Yan Feng, Jian Chen, Aijun Zhang

**Affiliations:** 1 US Department of Agriculture, Agricultural Research Service, Invasive Insect Biocontrol and Behavior Laboratory, Beltsville, Maryland USA; 2 US Department of Agriculture, Agricultural Research Service, National Biological Control Laboratory, Stoneville, Mississippi USA

## Abstract

Benzyl methyl ester, also known as methyl benzoate (MB), is a volatile organic compound that exists naturally as a floral fragrance in many plants. Our behavioral bioassays show that MB and some of its naturally occurring and synthetic analogs kill insects at different life stages. Compared to commercial pesticides containing pyriproxyfen and acetamiprid, MB and some analogs are 1.3 to 3.4 times more toxic to gypsy moth larvae and brown marmorated stinkbug nymphs. The arthropod repellent DEET is also a benzyl ester, and shares the same chemical skeleton with MB. They differ by the diethylamide ester and a methyl group on the benzene ring in DEET. However, unlike MB, DEET does not kill insects; instead, it deters or repels them. Exactly how DEET causes the repellent effect in target organisms is still a mystery. Due to the MB’s structural similarity to DEET, exploring the structure – activity relationship (SAR) of the MB analogs will provide useful information for the discovery of the mode and mechanistic actions of DEET as an insect repellent. In addition, the SAR will allow researchers to modify the chemical structure of the MB molecule, leading to the development of more efficient, safe, and environmentally – friendly green pesticides.

## Introduction

The world is facing unprecedented challenges in agriculture, with higher demands for food supplies and ever-increasing concerns on human health, food safety, and environmental sustainability^1,2^. Pesticides are substances used to control a variety of pests including insects and weeds; and they have become a paradox to human societies. Human populations were able to achieve enormous growth^3^ by using pesticides to protect people from insect-borne diseases like malaria^4^. On the other hand, pesticide use also posed great threats to human health and the environment. Currently the use of pesticides is ubiquitous. In the US, 80% of pesticides are used in agriculture with the remaining 20% being used in the non-agricultural sector^5^. Due to the overuse of pesticides, may insects have developed reisstances^6^. Pesticides are also harmful for species diversity and the environment. There is 42% fewer species of invertebrates in streams with severe pesticide contamination, and while many studies have been done there is still a need for further investigation into the ecosystem-wide effects of pesticide exposures^5,7^. The complete abandonment of pesticides is not possible, for at least the near future, so it is highly desired to develop environmentally benign but effective insecticide alternatives to the widely used toxic synthetic pesticides.

Some naturally occurring essential oils are believed to be more desirable as insect pest toxins than conventional synthetic insecticides due to their rapid environmental biodegradable property and potential lower toxicity to natural enemies, humans, and other mammals^8–10^. It has been reported that essential oils generally have quite favorable vertebrate toxicities^11^ and some of them were found to be 300 times to 3000 times less toxic to fish than some common synthetic insecticides, due in part to differing pharmacokinetics and detoxicative metabolism but may also be a result of a biorational mode of action^12^. Therefore, naturally occurring essential oils have great potential to be alternatives to synthetic pesticides^13–15^. Especially, many essential oil compounds have been using as food ingredients and are exempted from EPA registration; they are garnered more interest in recent years for developing commercial products in pest management. The essential oil based green pesticides often exhibit pesticidal activities to a broad spectrum of insects, and sometimes, due to the complex chemical components with multiple modes of action, they may have synergistic effects among constitutents^16^. Due to their high volatility in nature, essential oils are also important fumigants against agriculture and stored product insect pests^16^.

We have previously reported that the volatile organic compound (VOC) component, benzyl methyl ester, also known as methyl benzoate (MB), identified from fermented apple juice, exhibited sublethal or acute toxic effects against some insect pests, including the invasive fruit-infesting fly, spotted wing drosophila *Drosophila suzukii* Matsumura, brown marmorated stinkbug *Halyomorpha halys* Stål, diamondback moth *Plutella xylostella* L., and tobacco hornworm *Manduca sexta* L.^17^. In this study, another important invasive species, gypsy moth, *Lymantria dispar dispar* L., was also inspected for toxicity based on the availability of insect colony.

**Figure 1:**
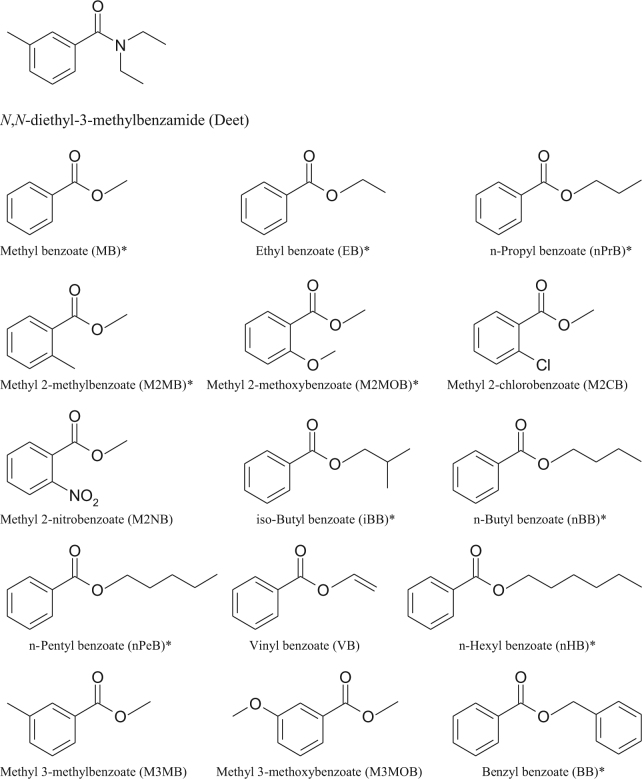
Chemical structures of DEET, methyl benzoate, and its analogs tested in this study. *Natural occurring volatile organic compound.

Besides MB, 14 methyl benzoate analogs, in which 9 of them are naturally occurring (Fig. 1, naturally occurring compounds were marked with asterisks), were assessed for their contact toxicities against the above insects. The structure – activity relationship (SAR) was also studied. For MB analogs, they were divided into two categories in such a way that one possessed different alcohol portions for evaluating the molecular size/dimension effect, including ethyl benzoate (EB), vinyl benzoate (VB), *n*-propyl benzoate (nPrB), *n*-butyl benzoate (nBB), *iso*-butyl benzoate (iBB), *n*-pentyl benzoate (nPeB), and *n*-hexyl benzoate (nHB). While the other had different substituents on the benzyl ring for examining the electrophilic/nucleophilic (electron-withdrawing/electron-donating) aromatic substitution influence, including methyl 2-methylbenzoate (M2MB), methyl 2-chlorobenzoate (M2CB), methyl 2-methoxybenzoate (M2MOB), methyl 2-nitrobenzoate (M2NB), methyl 3-methylbenzoate (M3MB), and methyl 3-methoxybenzoate (M3MOB). Other than above compounds, an aromatic benzyl ester instead of aliphatic ester, benzyl benzoate (BB) was also examined.

## Results

### Insecticidal activities against *D. suzukii*

**Figure 2:**
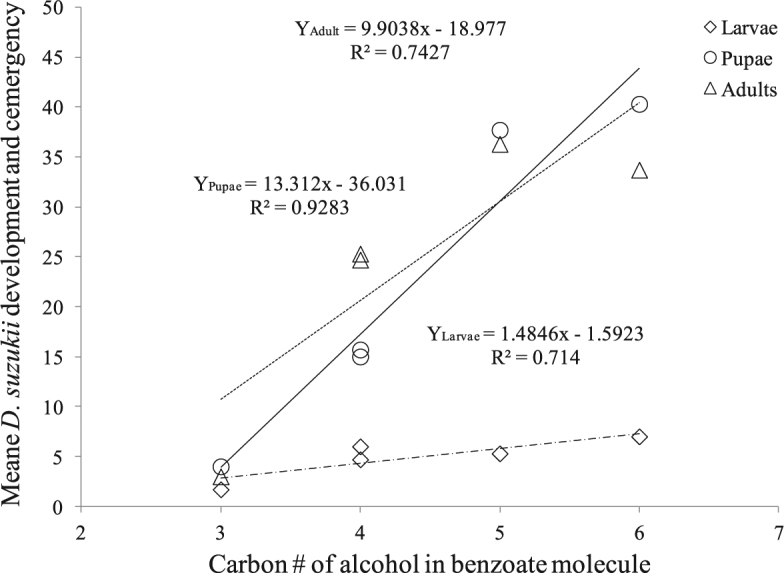
Correlation between toxicities and alkyl chain length of alcohols in benzoates against *D. suzukii*.

Among the 15 compounds tested, 9 of them exhibited potent toxicities against *D. suzukii* (Table 1). Direct application of compound at 1% concentration on 4 days pre-infested blue berries caused complete mortality of the *D. suzukii* with no larvae and pupae developed or adult flies emerging after 10 days of incubation at room temperature (Table 1). Toxicities were significantly affected by alkyl chain length/dimension of the alcohol portion of MB analogs. Although analog, nPrB, did not have the same efficacy compared with the 9 compounds listed in Table 1, it still showed good insecticidal activity against *D. suzukii*. However, when the carbon number in the alcohol portion of the compound is increased to more than three, the insecticidal activities were greatly decreased. Toxicity was negatively correlated with alkyl chain length (R² larvae = 0.9293; R² pupae = 0.9270; R² adult = 0.7739) (Fig. 2). While chain length of the alcohol increased to more than 4, nPeB and nHB did not show any insecticidal activities compared with the blank control (Table 1). The same poor toxicities were observed from two analogs with bulky alcohol dimensions, iBB and BB. On the other hand, aromatic substitution of MB did not affect the insecticidal activity at all. MB analogs with different functional groups (electron-withdrawing/electron-donating) on the benzene ring at 1% concentration showed the same potent toxicities as MB against *D. suzukii* (Table 1).

**Table 1:** Insecticidal activities of MB and analogs on larvae and pupae developments and adult emergences of D. suzukii^*^.

Treatment	Larvae^**^	Pupae^**^	Adults^**^
Control	6.7(0.9)	41.7(8.7)	35.7(7.9)
MB^***^	0	0	0
EB^***^	0	0	0
VB	0	0	0
M2MB^***^	0	0	0
M2MOB^***^	0	0	0
M2CB	0	0	0
M2NB	0	0	0
M3MB	0	0	0
M3MOB	0	0	0
nPrB^***^	1.7(0.3)	4.0(0.6)	3.0(0.6)
nBB^***^	4.7(1.2)	15.0(2.9)	25.3(1.5)
iBB^***^	6.0(1.5)	15.7(1.9)	24.7(0.9)
nPeB^***^	5.3(0.9)	37.7(6.7)	36.3(3.9)
nHB^***^	7.0(1.0)	40.3(7.0)	33.7(7.4)
BB^***^	5.7(0.7)	40.0(5.8)	40.0(8.4)

^*^100 berries pre-infested with 100 mixed-adult for 4 days/treatment, 50 berries were then soaked with 1% MB or other analog solutions and water control respectively for 2 min. Assessment was conducted after 10 days incubation at room temperature. ^**^Results are means of three replicates; numbers in parenthesis indicate the standard error. ^***^Natural occurring volatile organic compound.

### Nymphicidal effects against *H. halys*

Besides MB, 14 analogs and 2 commercial insecticides were tested against *H. halys* nymphs at the different life stages (Table 2). Similar results compared with *D. suzukii* were obtained; nPeB and nHB did not show insecticidal activities. Analogs with alkyl chain lengths less than three carbons resulted in LC_50_ values from 0.97 to 2.43 µL/vial. They were comparable to two commercial pesticides containing the active ingredients (AI) Acetamiprid and Pyriproxyfen (Table 3) tested in lab bioassays (LC_50_ values from 0.26 to 2.70 µL/vial) (Table 2). Once again, two analogs with bulky alcohol dimensions, iBB and BB, exhibited deficient toxicities against *H. halys* nymphs. All analogs with different nucleophilic aromatic substituents (electron-donating group) did not deeply affect the nymphicidal effects against *H. halys*, while analog with the electrophilic aromatic substituent (electron-withdrawing group) (M2NB) was ineffective as bulky esters iBB and BB (Table 2).

**Table 2:** Nymphicidal effects of MB and analogs against *H. halys* nymphs^*^.

Treatment	Stage	LC_50_ (95% CL) µL/vial	Slope ± SE
MB^**^	1^st^	1.03 (0.93–1.10)	7.69 ± 1.07
MB^**^	2^nd^	1.01 (0.86–1.12)	6.73 ± 1.11
MB^**^	3^rd^	1.23 (1.12–1.33)	5.28 ± 0.60
MB^**^	4^th^	2.39 (2.19–2.60)	6.10 ± 0.72
MB^**^	5^th^	1.77 (1.60–1.93)	6.00 ± 0.67
EB^**^	3^rd^	1.604 (1.468–1.756)	5.337 ± 0.591
VB	2^nd^	1.131 (0.757–1.337)	6.245 ± 1.155
M2CB	2^nd^	1.302 (1.137–1.610)	5.568 ± 0.849
M2MOB^**^	2^nd^	1.530(1.309–1.951)	4.393 ± 0.657
M2MB^**^	2^nd^	0.974(0.827–1.109)	4.165 ± 0.536
M3MOB	3^rd^	1.509(1.322–1.792)	3.549 ± 0.478
M3MB	4^th^	2.433(2.000–3.417)	3.606 ± 0.618
nPrB^**^	3^rd^	2.591(2.086–3.839)	3.427 ± 0.614
nBB^**^	4^th^	3.370(2.441–6.922)	2.752 ± 0.593
BB^**^	4^th^	5.619(3.028–44.599)	1.691 ± 0.485
iBB^**^	3^rd^	4.890(2.845–24.779)	1.821 ± 0.487
M2NB	3^rd^	n/a	3.175 ± 0.615
nPeB^**^	3^rd^	n/a	1.884 ± 0.809
nHB^**^	5^th^	n/a	1.679 ± 0.785
AP	2^nd^	0.257 (0.169–0.340)	2.078 ± 0.325
AP	3^rd^	0.475 (0.301–0.641)	1.750 ± 0.272
AP	5^th^	1.414 (1.165–1.642)	3.164 ± 0.394
PF	3^rd^	1.798 (1.397–2.797)	3.018 ± 0.450
PF	5^th^	2.700 (1.881–4.836)	2.315 ± 0.327

^*^270 nymphs are used for each treatment. MB data are copied from previous research^17^ for comparison purpose only. ^**^Natural occurring volatile organic compound. n/a means no concentration gradient was observed, therefore, it is not applicable by probit analysis using Polo Plus. AP is acetamiprid (the active ingredient of TriStar 8.5 SL Insecticide) and PF is pyriproxyfen (the active ingredient of Insect Growth Regulator).

**Table 3:** Commercially available pesticides tested in laboratory bioassay.

Trademark	Product	Active Ingredient (AI)	C%^*^
TriStar	8.5 SL Insecticide	Acetamiprid	8.5%
Distance	Insect Growth Regulator	Pyriproxyfen	11.23%

^*^Aqueous solution by weight.

### Ovicidal toxicities against *H. halys* and *P. xylostella*

The ovicidal toxicities of MB and analogs were evaluated by measuring hatch rate in a direct spray bioassay on *H. halys* and *P. xylostella* eggs. Analogs with alkyl chain lengths more than two carbons were ineffective against *H. halys* eggs, while substituents on the benzene ring did not deeply affect the ovicidal toxicities (Table 4).

**Table 4:** Ovicidal toxicities of MB analogs against *H. halys* eggs^*^.

Treatment	LC_50_ (95% CL) mg/cm^2^	LC_95_ (95% CL) mg/cm^2^	Slope ± SE
MB^**^	0.020 (0.012–0.026)	0.048 (0.036–0.090)	4.359 ± 1.108
EB^**^	0.014 (0.010 –0.019)	0.053 (0.036–0.105)	2.914 ± 0.556
VB	0.017 (0.010–0.024)	0.061 (0.042–0.122)	2.974 ± 0.620
M2CB	0.011 (0.008–0.013)	0.030 (0.022–0.059)	3.724 ± 0.816
M2MOB^**^	0.011 (0.006–0.015)	0.030 (0.020–0.111)	3.724 ± 0.816
M2MB^**^	0.014 (0.005–0.027)	0.39 (0.164–2.564)	1.144 ± 0.247
M2NB	0.010 (0.002–0.019)	0.067 (0.033–0.931)	2.028 ± 0.389
M3MB	0.016 (0.009–0.024)	0.097 (0.065–0.184)	2.128 ± 0.399
M3MOB	0.016 (0.008–0.026)	0.083 (0.051–0.189)	2.327 ± 0.566
nPrB^**^	n/a	n/a	0.571 ± 0.206
nBB^**^	n/a	n/a	0.369 ± 0.206
BB^**^	n/a	n/a	0.016 ± 0.213
iBB^**^	n/a	n/a	0.016 ± 0.214
nPeB^**^	n/a	n/a	0.184 ± 0.207
nHB^**^	n/a	n/a	0.033 ± 0.216

^*^270 eggs were used in each treatment. **Natural occurring volatile organic compound. n/a means no concentration gradient was observed, therefore, it is not applicable by probit analysis using Polo Plus.

**Figure 3:**
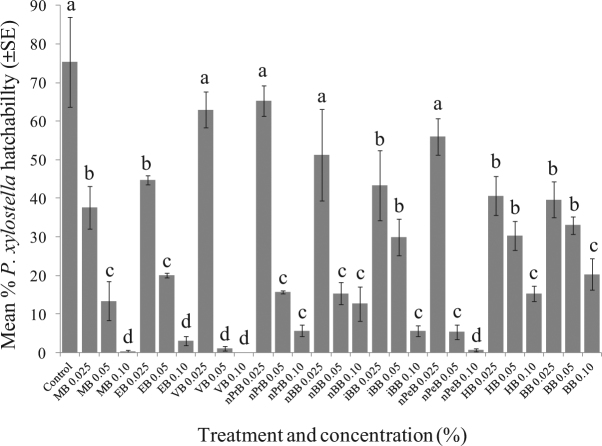
Ovicidal effects of selective MB and analogs against *P. xylostella* eggs (100 eggs were used in each bioassay). Hatchability was accessed after 10 days incubation at room temperature. Means followed by the different letters are significantly different at α = 0.05 (*N* = 3, *F* = 25.969; df = 27, *p* < 0.0001).

For *P. xylostella*, VB showed the highest toxicity. At 0.05% concentration, it exhibited significantly higher toxicity than that of MB and EB against *P. xylostella* eggs (Fig. 3). Surprisingly, nPeB with 5 a carbon chain length also showed comparable toxicity to MB at 0.1% concentrations (Fig. 3).

### Larvicidal toxicities against *L. dispar*

Again, VB showed the highest larvicidal toxicity against *L. dispar* (LC_50_ = 0.065), which was 3.4 and 1.3 times more toxic than the commercial pesticides containing the acetamiprid (LC_50_ = 0.221) and pyriproxyfen (LC_50_ = 0.086) (Table 5). It was also 1.7 times more potent than the MB. Once more, two analogs with bulky alcohol dimensions, nHB and BB, and one analog (M2NB) with electrophilic (electron-withdrawing) aromatic substitution, were not toxic.

**Table 5:** Larvacidal toxicities of MB and analogs against *L. dispar* larvae^*^.

Treatment	LC_50_ (95% CL) mg/cm^2^	Slope ± SE
MB^**^	0.114(0.091–0.134)	6.917 ± 0.852
EB^**^	0.115(0.107–0.123)	11.295 ± 1.545
VB	0.065(0.059–0.071)	10.082 ± 1.640
M2MB^**^	0.155(0.134–0.174)	6.671 ± 0.766
M2MOB^**^	0.230(0.170–0.294)	2.971 ± 0.487
M2CB	0.130(0.097–0.159)	7.291 ± 0.868
M3MB	0.102(0.083–0.121)	4.693 ± 0.540
M3MOB	0.234(0.2–0.258)	8.073 ± 1.126
nPrB^**^	0.159(0.135–0.199)	2.576 ± 0.402
nBB^**^	0.276(0.223–0.393)	2.264 ± 0.385
iBB^**^	0.193(0.165–0.234)	2.683 ± 0.386
nPeB^**^	0.165(0.118–0.208)	2.466 ± 0.354
PF	0.086(0.057–0.124)	2.429 ± 0.263
AP	0.221(0.167–0.291)	3.150 ± 0.419
M2NB	n/a	1.168 ± 0.545
nHB^**^	n/a	2.012 ± 0.843
BB^**^	n/a	2.858 ± 0.535

^*^270 larvae (1st stage) were used for each treatment. ^**^Natural occurring volatile organic compound. AP is acetamiprid (the active ingredient of TriStar 8.5 SL Insecticide) and PF is pyriproxyfen (the active ingredient of Insect Growth Regulator). n/a means no concentration gradient was observed, therefore, it is not applicable by probit analysis using Polo Plus.

## Discussions

A previous study from our lab showed that MB, a VOC identified from fermented apple juice, was a powerful green pesticide to several invasive insect species^17^. Current research demonstrates that MB analogs also exhibit some degrees of contact toxicity. Among 15 chemicals tested, 10 are natural occurring compounds (MB^18–24^, EB^25^, M2MB^26^, M2MOB^27^, nPrB^28^, nBB^29^, nPeB^30^, iBB^31^, nHB^21–24,26^, BB^21–24,32^) (Fig. 1), in which MB, EB, nPrB, M2MOB, iBB, and BB have been approved by the US Food and Drug Administration (21 CFR 172.515)^33^ and the European Union (EU Regulation 1334/2008 & 178/2002)^34^ for use as flavoring substances and adjuvants.

In nature, MB has been found to be used by flowers and insects as semiochemicals^18–20,35,36^. Besides the work of Feng and Zhang^17^, pesticidal property of MB has never been reported. This study demonstrated that MB and 14 analogs result in varying toxicities against four insect species, including *D. suzukii*, *H. halys*, *P. xylostella*, and *L. dispar*. For non-substituted MB analogs, toxicities against insects were deeply affected by molecular size/dimension. Generally speaking, the larger the ester moieties (longer carbon chain in alcohol or bulky alcohol, e.g. hexyl benzoate or benzyl benzoate), the lower contact toxicities were obtained. The insecticidal toxicities against *D. suzukii* larvae/pupae development and adult emergence, as well as larvicidal toxicities against *L. dispar* were negatively correlated with alkyl chain length and alcohol dimension. Ovicidal toxicity against *H. halys* disappeared when the alkyl group was increased to more than three carbons. However, the unsaturated ester (VB) revealed significantly higher toxicity than MB against *L. dispar* larvae and *P. xylostella* eggs.

Aromatic substitutions on the benzene ring in the MB molecule may also affect the toxicities of MB analogs against insects. Different electron-donating functional groups on the benzene ring did not greatly change the insecticidal toxicities against *H. halys* eggs and *D. suzukii* larvae/pupae development and adult emergence. However, the analog with an electrophilic (electron-withdrawing) aromatic substituent (M2NB) was totally ineffective against *L. dispar* larvae and *H. halys* nymphs.

Obviously, chemical structure of benzyl methyl ester is similar to the well-known insect repellent DEET, sharing the same chemical skeleton with the exceptions of the methyl ester (DEET is diethylamide ester) and *meta* methyl substitution. DEET remains the gold standard for current arthropod repellent testing^37^. It does not kill mosquitoes or other biting insects but deters or repels them and has been commonly used in combination with insecticides to strengthen their toxicity^38^. However, the exact mode of action of DEET in target organisms is still a mystery^39^, though it is believed to work by jamming the insect olfactory receptors^40–42^ or masking the smell of the host^43^. Our behavioral bioassays proved that the MB and its analogs could be taken up by insects via ingestion or contact at different stages. Our results also showed that the toxicities of MB analogs were correlated with alkyl chain length or dimension of the alcohol portion of MB analog molecules as well as being affected by aromatic substitution; especially, by electron-withdrawing group. Because of structural similarities of MB analogs and DEET, the discovery of MB and its analogs as insecticides via ingestion or contact and the understanding of the SAR of MB analogs may provide valuable information for further study of the mode and mechanistic actions of DEET as a mosquito and biting arthropod repellent. It also allows modification of the efficacy of methyl benzoate molecule by changing its chemical structure, enabling the development more efficient pesticides.

## Methods

### Chemicals

Methyl benzoate, Tween 20, Tween 80, ethyl benzoate, vinyl benzoate, *n*-propyl benzoate, *n*-butyl benzoate, benzyl benzoate, methyl 2-methylbenzoate, methyl 2-chlorobenzoate, methyl 2-methoxybenzoate, and methyl 2-nitrobenzoate were purchased from Sigma-Aldrich (St. Louis, MO). Compounds, *iso*-butyl benzoate, *n*-pentyl benzoate, and *n*-hexyl benzoate were purchased from Alfa Aesar (Tewksbury, MA). Methyl 3-methoxybenzoate (methyl m-anisate) and methyl 3-methylbenazoate (methyl *m*-toluate) were purchased from TCI America (Portland, OR). Acetone was used as solvent and purchased from Sigma-Aldrich (St. Louis, MO). All chemicals were used without further purification. Commercial pesticides: Distance insect growth regulator was purchased from Valent (Walnut Creek, CA), and TriStar 8.5 SL insecticide was purchased from Cleary Chemical (Alsip, IL). The active ingredients and corresponding concentrations for above commercial pesticides are listed in Table 3.

### Insects

The *H. halys* adults, nymphs, and eggs were obtained from colony maintained in the facility located at the USDA, ARS, Beltsville, MD. The *H. halys* colony was established in 2007 from adults collected in Allentown, PA, USA. Insects were reared on a diet of organic green beans and shelled sunflower and buckwheat seeds (2:1, w/w) in ventilated plastic cylinders and maintained in Percival incubator at 25 °C and 60% RH, under a 16 L:8D photoperiod^44^. Organic green beans were purchased from MOM’s organic market (College Park, MD, USA). Insect eggs were collected weekly and hatched in plastic Petri dishes with a water vial, and after molting to second-instars, the nymphs were transferred to ventilated plastic cylinders for the remaining fourth instars^20^. Adult males and females were separated 1 or 2 days after emergence and subsequently maintained in different containers.

The *P. xylostella* colony was reared and maintained on an artificial wheat germ diet^45^ at the same USDA facility. Eggs and larvae were put in closed cardboard cups (236 mL, 8.9 cm diameter, 5.7 cm height, Solo Cup Company, Lake Forest, IL) and kept in an incubator (Percival Scientific Inc, Perry, IA) at 25 °C, 34% RH, under a 16 L:8 D photoperiod in the same insectary. Adults were maintained in screened cage (30.5 cm × 30.5 cm × 30.5 cm, BioQuip Inc). Eggs were deposited on aluminum foil strips (approx. 5.0 × 30.5 cm) dipped in cabbage juice and collected after 3–4 days.

The *L. dispar* colony was reared and maintained on a simplified artificial wheat germ diet (one liter of diet contains 120 g wheat germ, 10 g USDA vitamin, 25 g casein, 8 g Wesson salts, 2.5 g sorbic acid, 1 g methylparaben, 15 g agar, and 825 g deionized water (DI) water at the same USDA facility. Egg masses were received from CPHST Otis laboratory (APHIS, Buzzard’s Bay, MA) on a monthly basis in a cardboard cylinder placed in a refrigerated carton, and kept in a refrigerator until used. Egg masses were stapled to the cardboard lids (Solo Cup, Lake Forest, IL). Larvae were kept in plastic cups (6 oz, Solo Cup, Lake Forest, IL) that have about 1 cm of diet poured into the bottom and closed with cardboard lids. Eggs and larvae were kept in an incubator (Percival Scientific Inc, Perry, IA) at 25 °C, 40% - 50% RH, under a 16 L:8 D photoperiod in the same insectary. Adults were not kept at the insectary. The larvae used in the bioassay were 1~2 days old in the first stage.

The *D. suzukii* colony was provided by Rutgers University. The colony was reared on cornmeal diet^46^ in polystyrene vials with plugs and kept in a Percival incubator at 25 °C, 34% RH, under a 16 L:8 D photoperiod in USDA, ARS, Beltsville facility. Blueberries (Cottle Farms, Cottle Strawberry Nursery, Inc, Faison, NC) used in insecticidal activity evaluation were purchased from MOM’s organic market, College Park, MD, USA.

### Laboratory bioassays

Bioassays were conducted in USDA Beltsville laboratory at 25 °C, 60% RH, under a 16 L:8D photoperiod with ∼1700-lux light illuminance. A fume hood was maintained at the same condition with face velocity at 129 FPM. Plastic cups (32 oz, diameter 4.5 inches, deep 5 inches) were purchased from papermart.com (CA). The cover had an 80 mm diameter hole cut into it with an 85 mm diameter mesh glued to it (mesh size, 81 × 81, BioQuip, CA). Polystyrene vials (height, 95 mm, diameter, 28.5 mm) and plugs were obtained from Fisher Scientific (Pittsburg, PA). The plastic cage (30 × 30 × 30 cm) was purchased from BugDorm (Rancho Dominguez, CA). Glass vial (20 mL), glass spray bottle (Amber glass with spray top, 30 mL), Petri dish (9 cm diameter), and Whatman filter paper (90 mm diameter) were obtained from VWR (Atlanta, GA). Deionized water containing 1% emulsifier (v/v), Tween 20 and Tween 80, at 1:1 ratio was used to make different VOCs water solutions and also used as blank control.

### Impacts of MB and analogs on *D. suzukii* control

To investigate the acute toxicity of MB analogs against *D. suzukii*, a published procedure was followed^47^. First, mixed-sex *D. suzukii* adults (100) were introduced into a plastic cage (30 × 30 × 30 cm) and reared on blueberries (100) for 4 days. The infested blueberries were then taken out from the cage. Half of the pre-infested blueberries (50) were dipped in100 mL aqueous emulsion of each analog at 1% concentration for 2 min as treatment, while the other half (50) were dipped in DI water for 2 min as blank control. After that, the corresponding blueberries were separately taken out, placed in two different Petri dishes, and allowed to air dry for 2 h. The treatment and blank control blueberries were then stored in two separate plastic cups (32 oz) with closed caps and incubated at room temperature for 10 days. The emergence of adults was then subsequently assessed. The development of larvae and pupae were further inspected by dissection of the treatment and blank control berries. Each treatment and blank control was repeated three times.

### Toxicities of MB, analogs, and commercial pesticides on *H. halys* nymphs

Bioassays were carried out in glass vials (20 mL), following a published procedure^48^. Filter paper was cut into round shaped pieces (2.4 cm diameter). A 50 µL acetone solution of one derivative or commercial pesticide with 9 different concentrations was loaded onto the filter paper evenly. The filter paper was dried for 1 min and then put into the bottom of vial. A small piece of green bean was placed onto the filter paper in the vial as a food source. Different life stages of *H. halys* nymphs were introduced into the vial and capped with a cotton ball. For each stage, total 30 nymphs were tested for each concentration. Because the size of nymph is different at different life stage, the number of nymph in the tested vial is varied. For the first instar nymphs, 10 nymphs were put into 1 vial. For the second and third instar nymphs, 5 nymphs were put into 1 vial. For the fourth instar nymphs, 3 nymphs were put into 1 vial. For the fifth instar nymphs, 2 nymphs were put into 1 vial. Mortality was assessed after 24 hr. Mortality data was subjected to probit analysis using Polo Plus for LC_50_ with 95% confidence interval calculation.

### Ovicidal toxicities of MB and analogs

The aqueous solutions with different concentrations of MB and varying analogs were separately stored in glass spray bottles according to a published procedure^47^. The eggs (10 for *H. halys* and 100 for *P. xylostella*) were laid on filter papers in Petri dishes. Eggs were sprayed with insecticidal solutions using the glass spray bottles (press spray bottle three times, ~ 0.5 mL) to completely cover the treatment areas. Then Petri dishes were covered with lids and maintained in a fume hood for 10 days. The Petri dishes were then inspected for presence of nymph/larvae development or numbers of unhatched eggs.

### Toxicities of MB and analogs on *L. dispar* larvae

Bioassays were carried out in plastic Petri dishes loaded with filter paper, following a published procedure^48^. Different concentration of the analog acetone solutions (600 µL) were applied to the filter papers (90 mm diameter) evenly. The papers were dried for 5 min in a fume hood to allow the acetone to completely evaporate and then put into the bottom of Petri dishes. 10 *L. dispar* larvae were put into each Petri dish and covered with a lid. 30 larvae were used for each treatment concentration (9 different concentrations). Mortality was assessed after 24 hr. Mortality data was subjected to probit analysis using Polo Plus for LC_50_ with 95% confidence intervals calculation.

### Data analysis

Comparisons of different treatments were analyzed using one-way ANOVA followed by Turkey-HSD test (KaleidaGraph, Synergy Software, for significance at α = 0.05). Polo Plus software (LeOra Software, Berkeley, CA) was used to conduct probit analysis for mortality data, and LC_50_ with 95% confidence intervals were estimated^49^.
